# P-971. Chogoria Rounds: A Novel Clinical Reasoning Tool for Residents

**DOI:** 10.1093/ofid/ofae631.1161

**Published:** 2025-01-29

**Authors:** Michael Leonard, Banks Kooken, Charles Barrier, Will smith

**Affiliations:** Carolinas Medical Center Atrium Health, Charlotte, North Carolina; Atrium Health Wake Forest School of Medicine, Charlotte, North Carolina; Atrium Health-Wake Forest School of Medicine, charlotte, North Carolina; University of Alabama Birmingham, Birmingham, Alabama

## Abstract

**Background:**

Teaching clinical reasoning requires a clinician being able to demonstrate their thought process when approaching a case, and a large volume of case exposure during residency to gain a basis of understanding. Training programs have observed improved application of clinical reasoning skills by internal medicine (IM) residents when a case-based reasoning curriculum is introduced. We present a novel educational tool for IM residents involving a series of case presentations (Chogoria Rounds, CR) from a physician partner in Kenya to general IM and Infectious Disease attendings, along with IM residents at different stages of training.

**Methods:**

Over an 18-month period, 30 cases were presented virtually, de novo, allowing attendings to discuss their clinical assessment of these cases in real time with the residents in attendance. A survey with Likert scale response (1 strongly disagree, 5 strongly agree) was distributed to residents to assess CR as an effective clinical reasoning teaching tool

**Results:**

Of the 30 presented cases, 11 were related to tuberculosis (pulmonary, miliary, meningitis), 10 to other infections (severe malaria, leishmaniasis) and other interesting cases such as African night shade ingestion. Thirteen residents were surveyed with 10 respondents. All 10 strongly agreed or agreed that CR was a useful clinical reasoning teaching tool (50% strongly agreed, 50% agreed), and 90% felt it impacted patient care. 80% reported CR drove them to read more about a particular disease process that was discussed. 50% of residents did not feel CR impacted their practice regarding high value care, but 90% felt it generated meaningful discussion surrounding resource utilization. All residents expressed interest in participating in a global medicine experience in the future, and 90% became more interested in global medicine.

**Conclusion:**

CR showed to be an effective clinical teaching tool for IM residents by giving them insight into clinical reasoning from expert attendings as well as rapid fire case conferences to provide increased volume of exposure.This virtual case conference model can easily be replicated for the benefit of residents and other learners at the US-based institution while at the same time benefiting those at the partnering international institution.

**Disclosures:**

**All Authors**: No reported disclosures

